# Influence of an MDP Salt–Based Surface Cleaner on Bond Strength to Saliva-Contaminated Dentin At Different Stages of a Self-Etch Adhesive Procedure: An In Vitro Pilot Study

**DOI:** 10.3290/j.jad.c_2503

**Published:** 2026-02-09

**Authors:** Ruhsan Müdüroğlu Adıgüzel, Leyla Kerimova Köse, Eda Çakmak Aktan, Neslihan Arhun

**Affiliations:** a Ruhsan Müdüroğlu Adıgüzel Assistant Professor, Department of Restorative Dentistry, Faculty of Dentistry, Başkent University, Ankara, Turkey. Investigation, methodology, writing – original draft, and preparation of the figures, read and approved the final version of the manuscript.; b Leyla Kerimova Köse Assistant Professor, Department of Restorative Dentistry, Faculty of Dentistry, Başkent University, Ankara, Turkey. Project administration, conceptualization, investigation, writing the manuscript, review, and editing, read and approved the final version of the manuscript.; c Eda Çakmak Aktan Assistant Professor, Department of Audiology, Faculty of Health Sciences, Başkent University, Ankara, Turkey. Consulted on and performed statistical evaluation, table preparation, read and approved the final version of the manuscript.; d Neslihan Arhun Professor, Department of Restorative Dentistry, Faculty of Dentistry, Başkent University, Ankara, Turkey. Conceptualization, investigation, methodology, proofread the manuscript, supervision, read and approved the final version of the manuscript.

**Keywords:** cavity cleaner, composite restoration, microshear bond strength, saliva contamination, self-etch adhesive

## Abstract

**Purpose:**

This *in vitro* study was designed to assess the influence of KATANA™ Cleaner (KC) on the microshear bond strength (μSBS) of composite resin restorations to saliva-contaminated dentin surfaces at various stages of the bonding process using a two-step self-etch adhesive system.

**Materials and Methods:**

A total of 140 sound human molars were randomly assigned into 14 groups according to the timing of saliva contamination and the decontamination approach. Saliva contamination was introduced at critical phases of adhesive application (before primer, after primer, after bond application, and after bond polymerization). KC was used for surface decontamination where applicable. Clearfil SE Bond adhesive and Filtek Z250 composite resin were applied using standardized procedures. μSBS test was performed after 24-h water storage, and failure modes were analyzed using stereomicroscopy. Additionally, dentin surfaces were evaluated under SEM. EDS was employed to evaluate the structure of the adhesive surface. Data were statistically analyzed using one-way ANOVA and LSD post hoc tests (α = 0.05).

**Results:**

A significant difference in μSBS was observed among the groups (P = 0.029). The highest μSBS (29.5 ± 9.25 MPa) was recorded in the group that was contaminated after primer application and subsequently decontaminated with KC prior to bonding, surpassing even the uncontaminated control group. Decontamination following contamination after polymerization also led to significantly improved μSBS (26.5 ± 10.78 MPa). Groups without any decontamination exhibited significantly lower μSBS. Predominantly cohesive (in composite resin) and mixed failure modes were observed in high-strength groups, while adhesive failures were more common in low-strength groups.

**Conclusion:**

KC significantly improved the μSBS of composite resin in saliva-contaminated dentin, particularly when applied after primer or post-polymerization contamination. These findings support the clinical use of KC as an effective decontamination protocol to maintain bonding performance under compromised isolation.

Recently, the growing popularity of composite restorations in restorative dentistry has sparked a surge in the development of advanced adhesive systems. Achieving high-quality adhesion and hybrid layer formation between the tooth structure and the composite resin is critical for the long-term success of restorations. However, factors such as moisture, blood, saliva, and oil contamination can impair adhesion quality and lead to clinical failures like microleakage, secondary caries, discoloration, and postoperative sensitivity.^3,16,19^ Furthermore, managing contamination becomes especially challenging in cases such as gingival inflammation or cervical carious lesions near the posterior region, often requiring additional time for the placement and polymerization of composite resins. Saliva contamination leads to the formation of an organic layer on the dentin surface that cannot be completely removed by rinsing with water.^6,21^ The timing of saliva contamination during the dentin bonding process significantly impacts the outcome of the adhesion,^1^ effective decontamination is required before placing composite resin restorations.^22^

While a well-isolated tooth surface is essential for good adhesion and saliva creates an unfavorable environment for ideal adhesion due to its water and glycoprotein content,^15,16^ recent research has shown variations in the influence of saliva contamination on adhesion with changes in the compositions, application steps, and modes of application of single and two-step self-etch bonding systems.^3,5,21^

It has been reported that contamination of dentin and the inner surface of indirect restorations by saliva or blood decreases the bond strength of adhesives.^10,12^ Some decontaminating agents for indirect restorations are either inadvisable for intraoral use or less effective in removing saliva contamination.^13^

Recently, an MDP salt-based cavity cleaner, KATANA™ Cleaner (KC; Kuraray Noritake Dental, Tokyo, Japan) was introduced. Created initially for cleaning zirconia ceramic surfaces, it contains a 10-methacryloxydecyl dihydrogen phosphate (10-MDP) salt derived from phosphate ester resin monomer, MDP, and triethanolamine.^20,22^ When applied to contaminated surfaces, KC binds to contaminants, weakening their surface tension and facilitating removal with water rinsing. The MDP monomer allows chemical reactivity with both zirconia and dentin/enamel, with a pH of 4.5, and unlike sodium hydroxide (NaOH) or potassium hydroxide (KOH), which are both caustic, KC is biologically compatible and can be used not only extraorally but also intraorally, making it an all-purpose universal cleaner for tooth structure and prosthetics.^14^

A recent study revealed that KC effectively neutralized the negative impact of saliva on the shear bond strength between resin and zirconia.^2^ However, there is a lack of scientific evidence in the literature regarding the effects of different adhesive strategies and current cleaning procedures on the bond strength of dentin contaminated with saliva. Therefore, this *in vitro* study aimed to evaluate the effect of KC on the microshear bond strength (μSBS) of composite resin restorations to dentin surfaces contaminated with saliva at various stages of the bonding process using a two-step self-etch adhesive system and to examine dentin surfaces under energy-dispersive X-ray spectroscopy (EDS) and scanning electron microscopy (SEM). The null hypothesis of the study was that the application of KC has no effect on μSBS of composite resin restorations contaminated by saliva at different stages of a two-step self-etch adhesive protocol (before/after primer application, after bond application, after bond polymerization) during the restoration placement process.

## MATERIALS AND METHODS

This study was approved by the Institutional Review Board and Ethics Committee of Başkent University (Project No: D-DA22/04) and supported by the Başkent University Research Fund. It adhered to the Modified CONSORT (Consolidated Standards of Reporting Trials) checklist.^9^

The need for informed consent was waived as the study utilized anonymized extracted human teeth obtained from an existing pooled collection. The use of these anonymized teeth without direct identifiers made it impracticable to obtain informed consent, and the waiver does not adversely affect the rights or welfare of the individuals.

### Sample Size Determination

The sample size was determined using the GPower 3.1 program by a biostatistician (EÇA). The effect size was determined as Cohen’s f = 0.531, an error level as α = 0.05, and a test power as (1-β) = 0.90, and the sample size calculation indicated that at least nine teeth per group would be sufficient to achieve the required statistical power.

### Sample Preparation

Table 1 shows the composition and the application steps of the adhesive system (Clearfil SE Bond, Kuraray Dental, Osaka, Japan), the cavity cleaner KC (KATANA™ Cleaner, Kuraray, Noritake Dental, Tokyo, Japan), and the composite resin (Filtek Z250, 3M ESPE, St. Paul, MN, USA) used in this research.

**Table 1:** Materials used, their corresponding composition, manufacturer, and application protocol

Material	Manufacturer	Composition	Application protocol
Clearfil SE Bond	Kuraray Noritake Dental Inc., Osaka, Japan	*Primer*: HEMA, 10-MDP, dl-Camphorquinone, water, hydrophilic aliphatic dimethacrylate. *Adhesive*: Bis-GMA, HEMA, MDP, colloidal silica, hydrophilic aliphatic dimethacrylate, dl-Camphorquinone, initiators, accelerators.	Apply primer for 20 s, mild air-blast. Apply bond for 20 s, mild air-blast, light-cure for 10 s.
Filtek Z250 (A1 Shade)	3M ESPE, St. Paul, MN, USA	*Matrix*: Bis-GMA, Bis-EMA, UDMA, TEGDMA Filler: zirconia, silica (0.01–3.5 μm), 78 wt%, 60 vol%.	Place restorative material at 2 mm thickness. Cure with a LED light curing device for 20 s.
KATANA™ Cleaner (KC)	Kuraray, Noritake Dental, Tokyo, Japan	Water, 10-MDP, triethanolamine, polyethylene glycol, stabilizer, dyes.	Application of Katana Cleaner (Kuraray Noritake) with a micro-brush to cover the area Rubbing for at least 10 s Thorough water rinsing until the color of the cleaner disappears and dry.
Bis-EMA: ethoxylated bisphenol A dimethacrylate; Bis-GMA: bisphenol A-glycidyl methacrylate; HEMA:2-hydroxylethyl methacrylate; MDP: 10- methacryloxydecyl dihydrogen phosphate; TEGDMA: triethylene glycol dimethacrylate; UDMA: urethane-dimethacrylate.

A total of 168 caries-free, extracted, and anonymized human molar teeth were collected from a pre-existing institutional tooth bank. Teeth were stored in distilled water at room temperature with water changed weekly until use. The teeth were cleaned, polished with pumice and rubber cups for 10 s, and then each tooth was embedded in autopolymerizing acrylic resin (Meliodent HC 1000 g 1 – Clear; Heraeus Kulzer, Germany) up to 1 mm below the cementoenamel junction.

**Fig 1:**
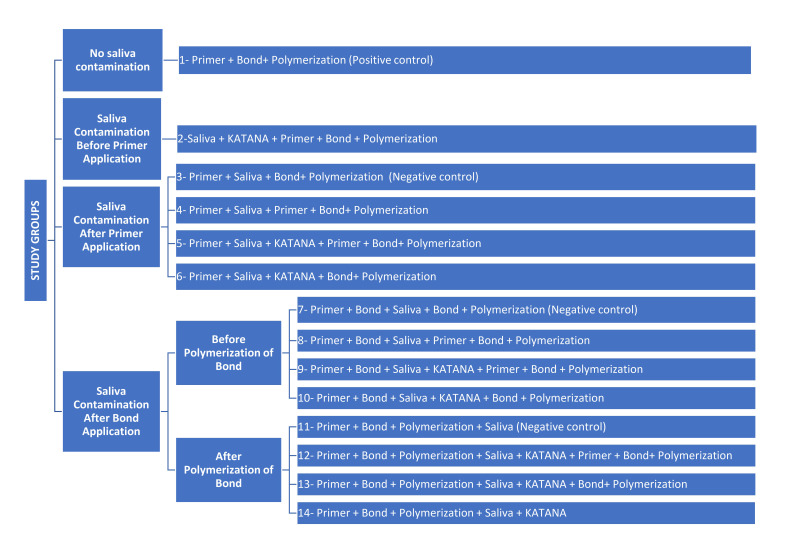
Schematic view of the study groups.

The occlusal surfaces of the teeth were cut horizontally to expose mid-coronal dentin using a low-speed water-cooled diamond saw in a cutting machine (Isomet, Bueler, IL, USA). The dentin surfaces were further standardized using 600-grit silicon carbide abrasive paper for 30 s to produce a uniform smear layer. The study comprised 14 experimental groups (140 teeth), categorized based on the timing of saliva contamination and subsequent surface treatments. Each of the 14 groups consisting of 10 teeth (n = 10) were randomly assigned per group. All of the materials were applied according to the recommendations of the manufacturer (Table 1). Saliva contamination and decontamination procedures of dentin were applied according to group-specific protocols. Additionally, two extra random teeth per group (n = 28) were used for the SEM and EDS analysis. All of the samples were prepared by a single operator blinded to the following test procedures (RMA). The flow chart of the experimental groups is presented in Figure 1.

#### Preparation of saliva-contaminated specimen groups

Unstimulated saliva was freshly collected from a healthy, non-smoking, and medication-free individual volunteer and used within 30 min to prevent degradation or contamination. The saliva was applied to the dentin surfaces using a microbrush for 60 s at various stages and then gently air-blown for 5 s. Following the saliva contamination, dentin decontamination procedures using KC was applied to the designated groups (G2,G5,G6,G9,G10,G12,G13,G14) by rubbing the surface with a microbrush for 15 s. The dentin surfaces were subsequently rinsed with water until the discoloration was no longer visible, according to the manufacturer’s instructions.

A two-step self-etch adhesive system (Clearfil SE Bond; Kuraray Dental, Osaka, Japan) was used for specimen preparation. Bond polymerization was performed following the manufacturer’s instructions using a 1200 mW/cm^2^ LED light-curing unit (Woodpecker LED.B; Guilin Woodpecker Medical Instrument Co., Guilin, Guangxi, China) verified with a calibrated radiometer before each session, with an 8 mm light guide tip diameter contact to surface for 10 s. For the μSBS test, a composite resin (Filtek Z250; 3M ESPE, St. Paul, MN, USA) was applied to the dentin surfaces of all groups using transparent plastic tubes with an internal diameter of 0.7 mm and a height of 2 mm, with one tube positioned per dentin surface, and fixed specimens were light-cured for 20 s using the same LED light source and the samples were stored at room temperature for 24 h in distilled water.

#### Microshear bond strength testing procedure (µSBS)

The prepared composite resin samples were placed in a universal μSBS testing machine (Z010, Zwick, Ulm, Germany) with a knife-edge chisel head and subjected to testing at a crosshead speed of 0.5 mm/min until failure. The µSBS was calculated by dividing the force obtained (in Newtons) by the restoration’s surface area (0.385 mm^2^) and recorded in MPa.

### Failure Mode Analysis

Failure modes after µSBS tests were examined under a stereomicroscope (SZ40, Olympus, Tokyo, Japan) at 15× magnification by a single operator (LKK) who was blinded to the contamination protocols. The failure mode was classified as “adhesive” if the fracture occurred at the interface between the composite resin and dentin, “cohesive” if within the composite resin or dentin, and “mixed” if it involved both the composite resin and dentin.

### Scanning Electron Microscopy (SEM) Analysis of Surface Morphology

Additionally, two more teeth from each group (a total of 28 teeth) were prepared without composite resin application. Each specimen was air-dried, mounted on metallic stubs, and sputter-coated with gold palladium. Morphological evaluation of dentin surfaces was performed using a SEM (QUANTA 400F Field Emission SEM, FEI, Hillsboro, Oregon, USA), accelerating voltage 15 kV, working distance 10 mm, spot size 3.0, and at magnification 2000x. Photomicrographs were also recorded. An operator who was blinded (NA) about the contamination protocols performed all the SEM evaluations.

### Energy-dispersive X-ray Spectroscopy (EDS) Analysis

The semiquantitative elemental microanalyzes were performed to evaluate the effect of decontamination protocols on adhesion by using energy-dispersive spectroscopy (EDS). Fourteen of the SEM specimens were evaluated for the EDS analysis. The EDS spectra were collected and analyzed using the EDAX Genesis software package (EDAX, Mahwah, NJ, USA), which was coupled with the Quanta 400F Field Emission SEM (FEI, Hillsboro, Oregon, USA) to enable elemental characterization for carbon (C), oxygen (O), calcium (Ca), fluorine (F), phosphorus (P), and magnesium (Mg). All outcome assessments were recorded and analyzed by a blinded operator (NA) to the group allocation.

### Statistical Analysis

Statistical analyses were conducted by IBM SPSS Statistics Version 25.0 (IBM, Armonk, NY, USA) by the blinded statistician to the procedures (EÇ). The Kolmogorov–Smirnov test was used to assess normality, and for homogeneity of variances, Levene’s tests were used. A one-way ANOVA test was conducted for group comparisons. The LSD post hoc test was applied for multiple comparisons with the control groups. A P value of < 0.05 was considered statistically significant for all analyses.

## RESULTS

The descriptive statistics of the μSBS values of the groups are summarized in Table 2. The identified outliers, which occurred in several groups because of pre-test failure, were excluded from the statistical analysis but were reported in Table 3 for transparency. A total of seven pre-test failures were observed across the experimental groups (Groups 1, 3, 4, 5, 10, 11, and 14). Each affected group had one specimen fail prematurely, corresponding to approximately 10% of the allocated samples. These pre-test failures were adhesive, occurring at the interface between the composite resin and dentin before testing.

**Table 2:** The descriptive statistics for the bond strength values of the groups

	Min–Max	Mean ± SD	F	P
G1 (n = 9) Primer + Bond+ Polymerization (Positive control)	17–29	21.44 ± 4.00	2.994	0.001*
G2 (n = 10) Saliva + KATANA + Primer + Bond + Polymerization	17–35	23.50 ± 6.06
G3 (n = 9) Primer + Saliva + Bond+ Polymerization (Negative control)	11–24	18.67 ± 4.27
G4 (n = 9) Primer + Saliva + Primer + Bond + Polymerization	17–35	24.44 ± 5.20
G5 (n = 9) Primer + Saliva + KATANA + Primer + Bond + Polymerization	20–31	24.22 ± 3.99
G6 (n = 10) Primer + Saliva + KATANA + Bond + Polymerization	22–53	29.50 ± 9.25
G7 (n = 10) Primer + Bond + Saliva + Bond + Polymerization (Negative control)	14–35	21.40 ± 6.02
G8 (n = 10) Primer + Bond + Saliva + Primer + Bond + Polymerization	14–34	20.10 ± 6.06
G9 (n = 10) Primer + Bond + Saliva + KATANA + Primer + Bond + Polymerization	15–23	18.00 ± 3.06
G10 (n = 9) Primer + Bond + Saliva + KATANA + Bond + Polymerization	15–35	22.89 ± 6.29
G11 (n = 9) Primer + Bond + Polymerization + Saliva (Negative control)	5–23	17.56 ± 5.66
G12 (n = 10) Primer + Bond + Polymerization + Saliva + KATANA + Primer + Bond+ Polymerization	16–47	26.50 ± 10.78
G13 (n = 10) Primer + Bond + Polymerization + Saliva + KATANA + Bond+ Polymerization	15–46	21.30 ± 9.52
G14 (n = 9) Primer + Bond + Polymerization + Saliva + KATANA	10–25	16.33 ± 4.97
SD: Standard deviation, F: One-way ANOVA, statistically significant differences mentioned with * in the last column (P < 0.05).

**Table 3:** Failure mode analysis

Groups	Adhesive	Cohesive (in composite resin)	Mixed	Pre-test failures (n, %)
Group 1 (n = 9)	0.00%	33.33%	66.67%	1 (10%)
Group 2 (n = 10)	0.00%	90.00%	10.00%	0 (0%)
Group 3 (n = 9)	11.11%	66.67%	22.22%	1 (10%)
Group 4 (n = 9)	0.00%	100.00%	0.00%	1 (10%)
Group 5 (n = 9)	0.00%	88.89%	11.11%	1 (10%)
Group 6 (n = 10)	10.00%	50.00%	40.00%	0 (0%)
Group 7 (n = 10)	0.00%	90.00%	10.00%	0 (0%)
Group 8 (n = 10)	0.00%	90.00%	10.00%	0 (0%)
Group 9 (n = 10)	0.00%	70.00%	30.00%	0 (0%)
Group 10 (n = 9)	0.00%	66.67%	33.33%	1 (10%)
Group 11 (n = 9)	11.11%	22.22%	66.67%	1 (10%)
Group 12 (n = 10)	0.00%	60.00%	40.00%	0 (0%)
Group 13 (n = 10)	0.00%	90.00%	10.00%	0 (0%)
Group 14 (n = 9)	11.11%	77.78%	11.11%	1 (10%)
[G1) Primer + Bond + Polymerization (Positive control), G2) Saliva + KATANA + Primer + Bond + Polymerization, G3) Primer + Saliva + Bond+ Polymerization (Negative control), G4) Primer + Saliva + Primer + Bond+ Polymerization, G5) Primer + Saliva + KATANA + Primer + Bond+ Polymerization, G6) Primer + Saliva + KATANA + Bond+ Polymerization, G7) Primer + Bond + Saliva + Bond + Polymerization (Negative control), G8) Primer + Bond + Saliva + Primer + Bond + Polymerization, G9) Primer + Bond + Saliva + KATANA + Primer + Bond + Polymerization, G10) Primer + Bond + Saliva + KATANA + Bond + Polymerization, G11) Primer + Bond + Polymerization + Saliva (Negative control), G12) Primer + Bond + Polymerization + Saliva + KATANA + Primer + Bond+ Polymerization, G13) Primer + Bond + Polymerization + Saliva + KATANA + Bond+ Polymerization, G14) Primer + Bond + Polymerization + Saliva + KATANA].

**Fig 2a to c:**
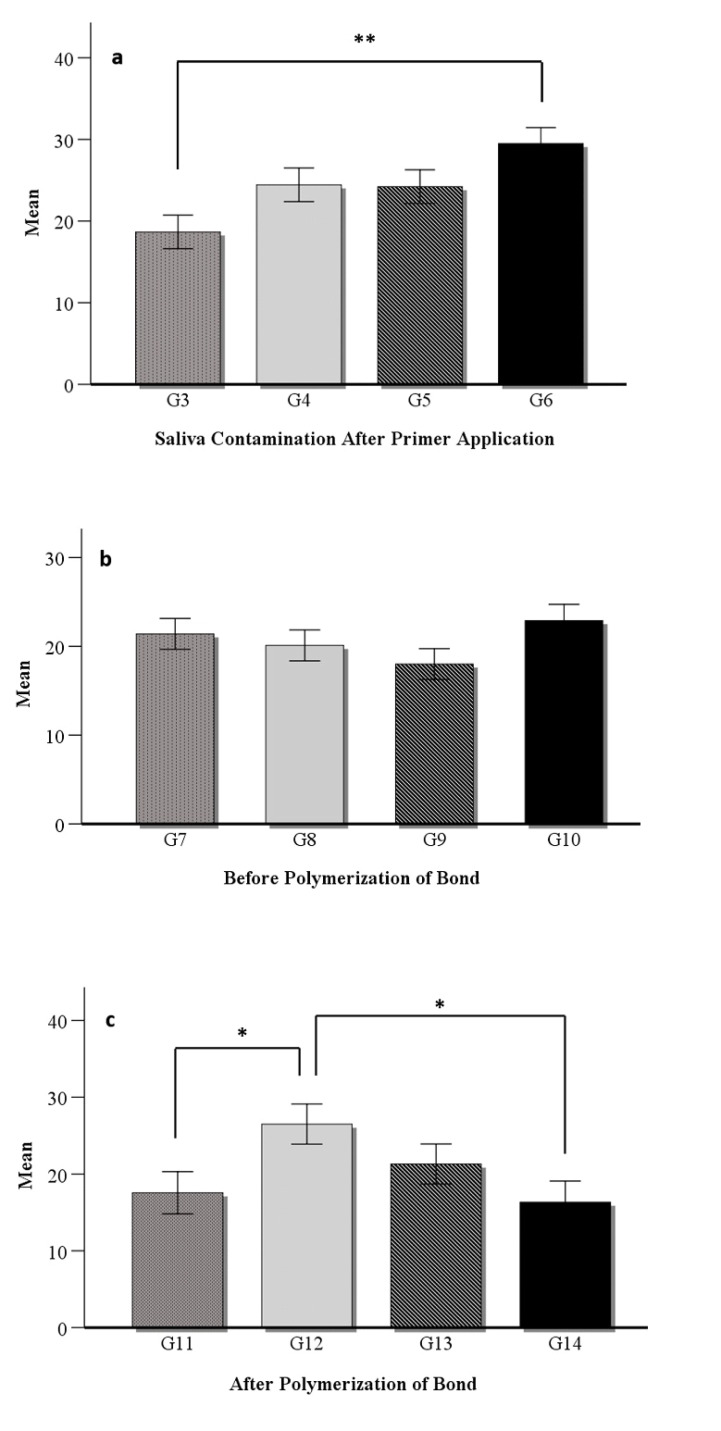
Graphs of bond strength values of all the contamination protocols (MPa): *(a)* Comparison of groups with saliva contamination after primer application [G3) Primer + Saliva + Bond + Polymerization (Negative control), G4) Primer + Saliva+ Primer + Bond+ Polymerization, G5) Primer + Saliva + KATANA + Primer + Bond+ Polymerization, G6) Primer + Saliva + KATANA + Bond+ Polymerization]. *(b) *Comparison of groups with saliva contamination before polymerization of bond [G7) Primer + Bond + Saliva + Bond + Polymerization (Negative control), G8) Primer + Bond + Saliva + Primer + Bond + Polymerization, G9) Primer + Bond + Saliva + KATANA + Primer + Bond + Polymerization, G10) Primer + Bond + Saliva + KATANA + Bond + Polymerization]. *(c)* Comparison of groups with saliva contamination after polymerization of bond G11) Primer + Bond + Polymerization + Saliva (Negative control), G12) Primer + Bond + Polymerization + Saliva + KATANA + Primer + Bond+ Polymerization, G13) Primer + Bond + Polymerization + Saliva + KATANA + Bond+ Polymerization, G14) Primer + Bond + Polymerization + Saliva + KATANA] (Statistically significant differences between groups mentioned with *P < 0.01 and **P < 0.001)

A statistically significant difference was found among the μSBS values (F = 1.974, P = 0.029). Multiple comparisons revealed a significant difference between the μSBS values of the G1 (positive control) and the G6 [(contamination after primer + KC (P = 0.008)], as well as between the G3 negative control (contamination after primer) and the G6 [(contamination after primer + KC (P < 0.001)] and G12 [(contamination after bond polymerization + KC + reapplication of primer and bond (P = 0.010)] groups. The highest μSBS was observed in G6 (29.5 ± 9.25). The μSBS measured in this group was found to be even higher than the positive control group, where no contamination occurred (Table 4). The abovementioned group was followed by G12, where the surface contaminated after polymerization was treated with KC, and both the bond and adhesive were reapplied (26.5 ± 10.78). The weakest μSBS was observed in G11 [contamination after bond polymerization (17.56)] and G14 [contamination after bond polymerization + KC (16.33)] (Table 2). Also, a statistically significant difference was registered between the G11 negative control and the G12 (P = 0.004). Figures 2a, 2b, and 2c display graphs of μSBS values of all the contamination protocols. However, significant differences were registered only in a limited number of scenarios (Table 3, P < 0.05). Generally, in groups where contamination occurred after the initial primer application and the surface was cleaned with either KC or the primer was reapplied (G4 = 24.44, G5 = 24.22, and G6 = 29.5), the μSBS was significantly enhanced compared to most other scenarios (P < 0.05).

**Table 4:** Multiple comparisons of the groups with control groups

Groups	Comparison	P value
**G1 (Positive control; n = 9)** Primer + Bond + Polymerization	G6	0.008*
**G3 (Negative control; n = 9)** Primer + Saliva + Bond + Polymerization	G6	< 0.001*
G12	0.010*
**G7 (Negative control; n = 10)** Primer + Bond + Saliva + Bond + Polymerization	G6	**0.007**
**G11 (Negative control; n = 9)** Primer + Bond + Polymerization + Saliva	G2	0.050*
G4	0.027*
G5	0.033*
G6	< 0.001*
G12	0.004*
LSD test, statistically significant differences are mentioned with * in the last column (P < 0.05). [G2) Saliva + KATANA + Primer + Bond + Polymerization, G4) Primer + Saliva + Primer + Bond+ Polymerization, G5) Primer + Saliva + KATANA + Primer + Bond+ Polymerization,G6) Primer + Saliva + KATANA + Bond+ Polymerization, G8) Primer + Bond + Saliva + Primer + Bond + Polymerization, G9) Primer + Bond + Saliva + KATANA + Primer + Bond + Polymerization, G10) Primer + Bond + Saliva + KATANA + Bond + Polymerization, G12) Primer + Bond + Polymerization + Saliva + KATANA + Primer + Bond+ Polymerization, G13) Primer + Bond + Polymerization + Saliva + KATANA + Bond+ Polymerization, G14) Primer + Bond + Polymerization + Saliva + KATANA].

### Failure Mode Analysis

The failure mode analysis of μSBS tests revealed a predominance of cohesive (in composite resin) and mixed failure types across all sample groups, with adhesive failures being infrequent. No adhesive failures were observed in most groups, except for G3, G6, G11, and G14, where adhesive failure rates ranged from 10.00% to 11.11% (Table 3).

Cohesive failure was the most frequently observed type in several groups, particularly in Groups G4 (100%), G2, G7, G8, and G13 (90% each), as well as G5 (88.89%). In contrast, mixed failures were more prevalent in G1 (66.67%) and G11 (66.67%), with other groups showing varying proportions.

### SEM Analysis of Surface Morphology

**Fig 3:**
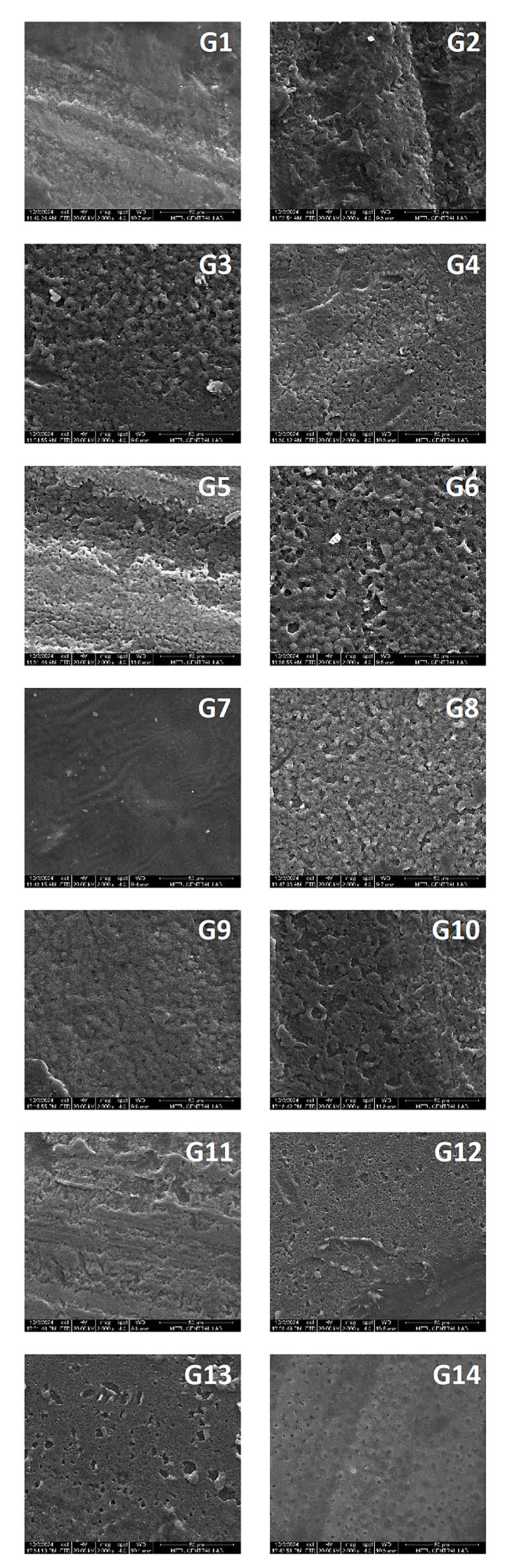
Representative SEM images of adhesive surfaces with different contamination protocols of all groups at 2000x magnification.

Figure 3 displays representative SEM images of the adhesive surface with different contamination protocols of all groups at 2000x magnification. Different levels of surface irregularities were observed depending on the surface treatments. Noticeable visual differences in topography were found between the positive control group (G1) and the other groups, with the smoothest surface observed in Figure 3. Rare surface irregularities were detected in the images of G7 (negative control). The most pronounced irregular surfaces were observed in G3 and G6.

#### Energy-dispersive X-ray spectroscopy analysis

Table 5 presents the representative semiquantitative chemical microanalysis of all tested groups (%). The lowest oxygen values were found in G1 and G7, and the highest was seen in G2. Groups G2, G4, G11, and G14 presented high Ca and P, whereas G1 and G2 presented high C values, indicating resin material content. G12 presented high Si and Ca values.

**Table 5:** The representative semiquantitative chemical microanalysis (%) of all tested groups

	G1	G2	G3	G4	G5	G6	G7	G8	G9	G10	G11	G12	G13	G14
C	80.24	12.62	53.30	46.47	59.22	62.64	78.31	57.23	59.19	58.45	–	–	62.84	62.38
O	17.48	39.80	23.59	22.32	23.59	24.29	17.87	22.59	24.59	25.28	47.63	53.40	24.22	18.98
Si	0.51	3.09	4.94	3.20	6.18	6.76	1.68	4.56	4.09	5.34	4.44	14.79	4.92	1.75
P	0.60	16.77	6.41	9.75	4.17	2.50	0.77	5.53	4.70	3.94	17.92	13.65	3.07	5.50
Ca	1.18	27.72	11.77	18.26	6.83	3.80	1.37	10.09	7.44	6.99	30.00	18.16	4.95	11.38
Total	100	100	100	100	100	100	100	100	100	100	100	100	100	100
[G1) Primer + Bond + Polymerization (Positive control), G2) Saliva + KATANA + Primer + Bond + Polymerization, G3) Primer + Saliva + Bond+ Polymerization (Negative control), G4) Primer + Saliva + Primer + Bond+ Polymerization, G5) Primer + Saliva + KATANA + Primer + Bond+ Polymerization, G6) Primer + Saliva + KATANA + Bond+ Polymerization, G7) Primer + Bond + Saliva + Bond + Polymerization (Negative control), G8) Primer + Bond + Saliva + Primer + Bond + Polymerization, G9) Primer + Bond + Saliva + KATANA + Primer + Bond + Polymerization, G10) Primer + Bond + Saliva + KATANA + Bond + Polymerization, G11) Primer + Bond + Polymerization + Saliva (Negative control), G12) Primer + Bond + Polymerization + Saliva + KATANA + Primer + Bond+ Polymerization, G13) Primer + Bond + Polymerization + Saliva + KATANA + Bond+ Polymerization, G14) Primer + Bond + Polymerization + Saliva + KATANA] (C: Carbon, O: Oxygen, Si: Silicon, P: Phosphorus, Ca: Calcium).

## DISCUSSION

The objective of the present study was to evaluate the μSBS after treating dentin surfaces with an MDP salt-based cavity cleaner (KC), contaminated at different stages with saliva, before application of composite resin restorations. This *in vitro* study aimed to assess the effectiveness of dentin decontamination under different conditions, rather than provide a broad product comparison across multiple commercial brands. The findings of the present study revealed that the μSBS to dentin in G6 (Primer + Saliva + KATANA + Bond+ Polymerization) was higher than that in the control group. Therefore, the null hypothesis suggested that the application of KC has no effect on the μSBS of composite resin restorations contaminated by saliva at different stages of a two-step self-etch adhesive protocol (before/after primer application, after bond application, after bond polymerization) was partially rejected.

KC contains a 10-MDP triethanolamine salt, which functions as an amphiphilic surfactant designed to facilitate the removal of hydrophobic contaminants. Given its pH of 4.5 and the absence of an alkaline cleaning mechanism, KC is considered safe for intraoral use. Thus, it may be considered for clinical situations when dental professionals need to apply a decontaminant material prior to restoration placement. This finding is consistent with the results of a limited number of previously conducted studies, which demonstrate that KC is effective in enhancing bond strength to dentin.^6,17^

A recent study by Etiennot et al^8^ focused on the effectiveness of 10-MDP-based decontamination agents (KC and Clearfil SE Bond 2 primer) on saliva-contaminated dentin using both one- and two-step adhesive systems, with bond strength measured both immediately and after artificial aging. Differently, our study investigated KC effect across different contamination stages (before/after primer, after bond application, post-polymerization) using only a two-step adhesive system, and evaluated µSBS without aging protocols. Also, surface analysis of specimens under SEM, and EDS analyses were done. Both Etiennot et al’s and our study emphasized the importance of decontamination of saliva-contaminated dentin. The referenced study^18^ reported that Clearfil SE Bond 2 performed well even without additional decontamination, while decontamination was necessary to improve the bonding performance of the universal adhesive. In our study, the use of KC significantly improved μSBS, particularly in Groups G6 and G12. This suggests that while some adhesives may tolerate contamination without additional treatment, applying a decontamination step like KC can further enhance bonding performance.

The distinctive chemical structure of 10-MDP, characterized by hydrophilic and hydrophobic groups positioned at opposite ends, enables it to interact effectively with saliva and other contaminants, disrupting their adhesion to dental surfaces.^20,23^ 10-MDP’s dual roles in both decontaminating and enhancing adhesion through its amphiphilic structure, forming micelles that help remove contaminants without compromising bonding. Consistent with this, our study showed that applying KC after contamination (G6 and G12) significantly improved μSBS, even surpassing the uncontaminated control in G6. These findings experimentally support the proposed mechanism, confirming that 10-MDP not only cleans but also strengthens the adhesive interface.

Saliva contamination has different effects at various stages of the bonding procedure^15^, so decontamination options should be applied according to the stage at which contamination occurred. In previous studies, saliva contamination reduced the bond strength of Clearfil SE Bond before the application of primer.^7,11^ However, no surface cleaner was applied in these studies. Another study investigating the effect of contamination at this step reported that the bond strength did not differ significantly between the saliva-contaminated group (whether a cleaner was applied or not) and the control group.^23^ The results of the current study were partially consistent with the findings of the study published by Sheikh et al regarding studies on the reapplication of primer,^19^ Hiraishi et al reported that re-priming contaminated dentin restored the bond strength to values comparable to the control (non-contaminated) group, but only rinsing before re-priming resulted in a reduction in bond strength.^11^ In the present study, rinsing and drying of the contaminated surface were performed before the reapplication of primer, and no difference was found compared to the control group. Moreover, cohesive failure (in composite resin) was the predominant failure mode, in groups primarily involved saliva contamination before primer application with subsequent primer reapplication (Group 4) or the use of KC (Groups 2, 5, 7, 8, and 13), suggesting that these approaches may have enhanced bond integrity. The absence of adhesive failure in these groups suggests that reapplying primer or incorporating KC helped maintain bonding strength despite contamination. Consequently, based on the findings of this study, the use of KC on dentin surfaces contaminated before primer application can be recommended to clinicians.

The chemical adhesion and dissolution of the adhesive systems are influenced by the stability of the calcium salt formed after progressing the interaction between the functional monomer and calcium in hydroxyapatite. The 10-MDP monomer, found in Clearfil SE Bond, interacts with hydroxyapatite and produces a very stable bond, releasing a low dissolution rate of its calcium salts in water. However, it should be considered that besides the bonding potential of functional monomers, the performance of adhesives is also related to the applied substrate.^5^ Previously published studies documented that in self-etch adhesives, saliva contamination is critical after primer application, as contaminant deposits can impair monomer diffusion, leading to decreased adhesion.^7,15,18^ The surface energy of the primed tooth surface contaminated with saliva decreases as an interaction of calcium phosphate structures with the proteins from saliva and dentin can deposit and form a physical barrier, weakening infiltration of bonding agent.^15^

In the previous studies, it is reported that a decrease in the bond strength of a two-step self-etch adhesive system (Clearfil SE Bond 2 and Clearfil SE Bond) is observed in post-primer contamination and decontaminating the surface by rinsing, drying, and reapplying the primer and adhesive considerably rehabilitated the SBS.^15,18^ Similarly, a recent systematic review by Bourgi et al^4^ recommended decontamination methods, such as rinsing with water spray and reapplying the bonding system, can reverse the negative effects caused by saliva or blood contamination. These findings align closely with our study’s outcomes. However, Vieira et al^24^ reported that saliva contamination consistently weakened the bond strength of Clearfil SE Bond regardless of the stage of the application and further noted that water rinsing and primer reapplication were ineffective in restoring the reduced bonding performance. These findings partially contradict the results of the current study.

In the current study, it was observed that the μSBS decreased as a result of saliva contamination that occurred after the application and polymerization. This decrease was statistically significant when compared to the μSBS of the before-primer-contaminated group but was insignificant compared to the control (non-contaminated) group.

Saliva contamination after polymerization of bonding agents before composite resin can decrease the quality of adhesion, and there are controversial findings regarding rehabilitating bond strength by using decontaminants. A previous study reported that decontamination by rinsing, drying, and reapplying the adhesive after curing did not seem to add any benefit to the bond strength of self-etching adhesives.^15^ On the other hand, Cobanoglu et al. observed that repeating the bonding steps after the decontamination procedure of the bonding agent contaminated with saliva may recover the bonding capacity of Clearfil SE Bond, and this finding correlates with the results of the present study.^7^ According to the findings of the failure mode analysis of the present study, it could be suggested that the bonding performance depended on the timing of saliva contamination.

Overall, the statistical results of μSBS and failure mode analysis were largely consistent. Groups with higher μSBS showed more cohesive (in composite resin) failures, while those with lower μSBS tended to exhibit more mixed and adhesive failures, except in Groups 6 and 11. Interestingly, some lower-strength groups had more cohesive failures(in composite resin) than the group with the highest μSBS. This may be attributed to the material itself, suggesting that even when adhesion is compromised, failure doesn’t always occur at the adhesive interface, possibly due to substrate factors. Pre-test failures were also noted in several groups, and they were adhesive failures. Although they were excluded from statistical analysis, their occurrence highlights the clinical vulnerability of the dentin surfaces. It should also be considered that some variability in dentin substrate quality (such as permeability, pulpal fluid flow, mineralization, sclerotic areas, and carious regions) may have contributed to these failures.

The results of this study highlight that saliva contamination does not uniformly compromise adhesion but rather its impact depends on the timing within the bonding protocol. Clinically, this suggests that when contamination occurs before primer application, additional steps such as reapplication of primer or the use of KC are essential to maintain bond integrity. Conversely, contamination occurring after polymerization may not require complex interventions in every case. These findings may assist clinicians in prioritizing and tailoring decontamination strategies during restorative procedures, especially under time-sensitive or challenging clinical conditions.

An interesting observation was that the μSBS in G6 exceeded that of the uncontaminated control. This finding suggests that KC may not only compensate for the negative effects of contamination but also potentially enhance bonding under certain conditions. While this is promising, it should be interpreted with caution, as *in vitro* results may not fully predict long-term intraoral performance. Nevertheless, this result opens the possibility that some decontamination agents could serve both corrective and enhancing roles within adhesive dentistry.

There are several limitations in this study that should be considered. The first limitation is the mandatory exclusion of current values in the negative control group due to premature failure. The second limitation is that the study examined only saliva contamination, which may occur at different stages during the application of a two-step self-etch adhesive system. In clinical practice, contamination scenarios can vary widely with blood, saliva, and oil, and dentists use different adhesive systems. The use of a single adhesive system, cleaner, and composite resin was intentional to control material-related variability and focus on the effect of saliva contamination and decontamination protocols. However, this may limit generalizability, which is acknowledged as a study limitation. Besides, unstimulated saliva was chosen to better mimic clinical contamination, while acknowledging variability in composition between individuals. Therefore, further studies are needed with different contamination models and adhesive systems, which should include thermocycling and long-term water storage to assess the MDP salt-based cleaner’s long-term stability. Finally, comparing the results of the present study with those of previously published studies is challenging because of differences in study designs.

## CONCLUSION

Considering the limitations mentioned, it can be concluded that an MDP salt-based cavity cleaner (KC) could be applied to contaminated surfaces to enhance bond strength, especially when contamination occurs before primer application. Also, no statistically significant reduction in μSBS was observed after saliva contamination regardless of whether contamination occurred (after primer or bond application steps). The study results also demonstrated that saliva contamination at different stages of adhesive application significantly influences failure modes, with early-stage contamination having the most detrimental impact. Nevertheless, reapplication of primer or the use of KC appeared to mitigate the negative effects of contamination.

### Clinical Relevance

Saliva contamination during early adhesive application steps significantly increases failure risk, but reapplication of primer or using KATANA™ Surface Cleaner may be helpful to mitigate the negative effects of contamination.

### Acknowledgments

We would like to thank the research assistants in our department for helping with experiments.

#### Funding statement

This research was supported by the Başkent University Research Fund (Grant No: D-DA22/04). It did not receive any specific grant from funding agencies in the public, commercial, or not-for-profit sectors.

#### Competing interests

The authors declare that they have no conflicts of interest or competing interests.

#### Availability of data and materials

The datasets used and/or analyzed during the current study are available from the corresponding author on reasonable request.

#### Ethics approval and consent to participate

This study was approved by the Institutional Review Board and Ethics Committee of Başkent University (Project No: D-DA22/04). All procedures performed in the study involving human material were in accordance with the 1964 Helsinki Declaration and its later amendments or comparable ethical standards.

#### Declaration of generative AI and AI-assisted technologies in the writing process

During the preparation of this work, the authors used ChatGPT in order to improve the readability and language of the manuscript. After using this service, the authors reviewed and edited the content as needed and took full responsibility for the content of the published article.
